# Driving polar growth

**DOI:** 10.7554/eLife.57043

**Published:** 2020-05-07

**Authors:** Neeraj Dhar

**Affiliations:** Global Health Institute, Ecole Polytechnique Fédérale de LausanneLausanneSwitzerland

**Keywords:** peptidoglycan, mycobacteria, corynebacteria, bacteria, Other

## Abstract

Profiling the phenotype of 200,000 mutants revealed a new cofactor that may help a group of rod-shaped bacteria elongate and grow.

**Related research article** Sher JW, Lim HC, Bernhardt TG. 2020. Global phenotypic profiling identifies a conserved actinobacterial cofactor for a bifunctional PBP-type cell wall synthase. *eLife* **9**:e54761. doi: 10.7554/eLife.54761

Bacteria come in a variety of shapes and sizes – some are round, some are spiral and some are rod-shaped. The mechanisms that bacteria use to generate and maintain these diverse shapes as they grow is an area of active research (Kysela et al., 2016). The cell wall of bacteria contains a net-like structure called peptidoglycan, and it is thought that this structure maintains the shape of the cell (Egan et al., 2017). As bacteria grow, nascent peptidoglycans and other materials are inserted into the cell wall in a complicated process involving multiprotein complexes that contain various enzymes (Höltje, 1998; Pazos et al., 2017).

The process of cell wall growth has been widely studied in rod-shaped bacteria such as *Escherichia coli* and *Bacillus subtilis*, which grow by adding new material to the long sidewalls of the cell rather than to the ends (Daniel and Errington, 2003). But not all rod-shaped bacteria grow this way. For example, bacteria belonging to the Actinobacteria phylum – which includes the pathogens that cause tuberculosis, leprosy, and diphtheria – grow by adding new material to the ends (or poles) of the cell (Kieser and Rubin, 2014; Cameron et al., 2015).

It has been suggested that scaffold proteins and intermediate filaments target the machinery that synthesizes peptidoglycans to the cell poles, in order to restrict growth to this region (Letek et al., 2008; Fiuza et al., 2010). However, many aspects of polar growth, including the composition of the enzyme complexes and the cofactors involved, are still unknown. Now, in eLife, Joel Sher, Hoong Chuin Lim and Thomas Bernhardt from Harvard Medical School report how a newly discovered cofactor localizes a peptidoglycan synthase enzyme to the poles of bacterial cells (Sher et al., 2020).

To identify the components involved in polar growth, Sher et al. studied a library of 200,000 mutants of *Corynebacterium glutamicum* (a member of the Actinobacteria phylum) in which each strain is mutated for a specific gene or pathway. The bacteria were exposed to various stressful conditions or antibiotics, and a phenotypic profile was generated for each mutant based on how they responded. Further analysis revealed that genes which have a similar role, or work together in the same pathway, exhibit similar characteristics when mutated. This allowed Sher et al. to identify which genes are involved in polar growth.

Sher et al. studied the behavior of a previously unknown gene that codes for a protein named CofA. The phenotypic profile of CofA mutants was highly correlated with the profiles of strains carrying a genetic mutation in an enzyme called PBP1a, which synthesizes peptidoglycans. Fluorescent tagging revealed that CofA was localized at the cell poles of *Corynebacterium*, which is consistent with previous studies that found peptidoglycan synthases (such as PBP1a) to also be located at this region (Valbuena et al., 2007).

Individually deleting the genes that code for CofA and PBP1a showed that these two proteins depend on each other for their localization. Further experiments revealed that CofA acts as a cofactor and binds to the transmembrane domain of PBP1a, helping the enzyme accumulate at the tips of the cell.

Paralogs of the gene coding for CofA and its transmembrane domains are found throughout the Actinobacteria phylum. Sher et al. showed that CofA proteins in pathogenic bacteria, such as *C. jeikium* and *M. tuberculosis*, also interacted with their PBP1a counterpart in a specific manner. The CofA protein in *M. tuberculosis* was found to have an extended N-terminal cytoplasmic domain and deleting this region facilitated the interaction between CofA and PBP1a. However, the role of this N-terminal domain was not investigated further.

This study is the first to identify a conserved cofactor that modulates the behavior of peptidoglycan synthases in Actinobacteria. It also raises several tantalizing questions: Would removing CofA cause the growth of *Corynebacterium* to be less polar? Does deleting CofA and PBP1a change how peptidoglycan units are inserted into the cell wall? It would also be useful to mine the phenotypic profiles of other mutants to see if there are other unidentified cofactors of the PBP proteins.

Several disease-causing pathogens use this mode of polar growth, which is why it is important to study the components and mechanisms involved. The bacterial cell wall is repeatedly used as a target for antibiotic development. These findings could identify new drug targets, which may help combat the rising rates of antibiotic resistance, especially in the case of tuberculosis. Furthermore, the phenotype profiling approach used by Sher et al. could be used to determine the role of previously uncharacterized proteins and identify which proteins and genes participate in the same biological pathway.
